# Engineered T lymphocytes eliminate lung metastases in models of pancreatic cancer

**DOI:** 10.18632/oncotarget.24122

**Published:** 2018-01-10

**Authors:** Qiang Sun, Shixin Zhou, Jingjing Zhao, Changwen Deng, Ruidi Teng, Yiding Zhao, Jiajia Chen, Jiebin Dong, Ming Yin, Yun Bai, Hongkui Deng, Jinhua Wen

**Affiliations:** ^1^ Department of Cell Biology and Stem Cell Research Center, School of Basic Medical Sciences, Peking University Health Science Center, Beijing, China; ^2^ Beijing Vitalstar Biotechnology Co., Ltd., Beijing, China; ^3^ The MOE Key Laboratory of Cell Proliferation and Differentiation, College of Life Sciences, Peking-Tsinghua Center for Life Sciences, Peking University, Beijing, China; ^4^ Shenzhen Stem Cell Engineering Laboratory, Key Laboratory of Chemical Genomics, Peking University Shenzhen Graduate School, Shenzhen, China

**Keywords:** adoptive cell therapy, mesothelin, CAR, lung metastasis

## Abstract

Pancreatic cancer is known as one of the most lethal cancers in the world. A majority of advanced stage pancreatic cancer patients are diagnosed with distant metastasis and given poor prognoses, calling for a better therapeutic option. Mesothelin, which is overexpressed in pancreatic cancer and other solid tumors, is a potential target for pancreatic cancer immunotherapy. Adoptive transfer of T cells engineered with chimeric antigen receptors (CART cells) was effective for treating CD19-positive leukemia, but it is more difficult for CART cells to eliminate solid tumors. Because distal metastasis is an important malignant behavior of solid tumors, we investigated whether meso-CART cells exert anti-tumor effects against distant metastases. After expressing meso-CAR in human primary T lymphocytes, the resultant meso-CART cells released cytokines in response to and exhibited cytolytic effects on mesothelin-positive tumor cells *in vitro*. Injection of meso-CART cells into tumor-bearing mice moderately delayed subcutaneous tumor growth and eliminated lung metastases. This is the first study to show that meso-CART cells are effective against lung metastases induced by intravenous injection of pancreatic tumor cells. Our results suggest that meso-CART cells may be an effective clinical treatment for mesothelin-positive primary and metastatic tumors in pancreatic cancer patients.

## INTRODUCTION

Pancreatic cancer is the seventh leading cause of death worldwide and is highly lethal, with 1- and 5-year survival rates of 27% and 7%, respectively [1, 2]. The major cause of poor prognosis in pancreatic cancer is distant metastasis, in which primary cancer spreads to normal organs such as the liver, lung, abdominal lymph nodes, or other distant organs [3, 4]. Distant metastasis is frequently observed even in patients with early-stage pancreatic cancer. The standard treatment for post-resection pancreatic cancer patients is low-dose chemotherapy combined with radiotherapy. However, these therapies extend patient survival by less than one year and have limited therapeutic effects [5], indicating that new therapeutic paradigms for pancreatic cancer and associated metastatic lesions are urgently needed.

Mesothelin is a tumor-associated antigen that is overexpressed on tumor cell membranes in a variety of human cancers, including mesothelioma, pancreatic cancer, ovarian cancer, and lung adenocarcinomas [6–9]. The expression of mesothelin on tumor cells plays an important role in tumor growth, survival, metastasis, and progression [10]. Mesothelin may therefore be an effective target for cancer immunotherapy.

In recent years, adoptive cell therapy, particularly with chimeric antigen receptor-engineered T cells (CART cells), has shown great potential as a cancer immunotherapy [11, 12]. The chimeric antigen receptor (CAR) consists of a single-chain fragment variable (scFv) that targets tumor antigens, a transmembrane region, a hinge region, and an intracellular signal domain [13–15].

Several preclinical studies have examined the efficacy of treatment with CART cells that target mesothelin (meso-CART cells). Lentivirus-transfected meso-CART cells reduced the size of, and ultimately eliminated, pre-established subcutaneous tumors [16]. In addition, multiple injections of RNA-transfected meso-CART cells repressed the growth of subcutaneous and intraperitoneal (i.p.) human-derived tumors [17]. Recently, a fully-human anti-mesothelin scFv was incorporated into meso-CART cells to overcome the transgene immunogenicity that resulted from the use of murine scFv; these cells also promoted regression of large tumors [18]. In a phase I clinical trial in mesothelioma patients, Beatty *et al.* reported that meso-CART cells transiently expressed in peripheral blood migrated to primary and metastatic lesions, where they exerted limited antitumor effects [19]. Although several preclinical studies have demonstrated the antitumor effects of meso-CART cells in primary or i.p. tumors, there are no effective treatments for pancreatic cancer-induced lung metastases in advanced stage disease. Moreover, few preclinical studies have examined the efficacy of meso-CART cells in treating lung metastasis in pancreatic cancer patients. The therapeutic effects of meso-CART cells in primary pancreatic cancer and metastatic lung lesions should therefore be evaluated further. Because metastasis is primarily a result of distal colonization by circulating tumor cells, we induced the development of lung metastases here with i.v. injections of tumor cells to mimic metastases arising from a primary tumor lesion.

In this study, we designed a meso-CAR consisting of CD8α signal peptide, anti-mesothelin scFv, a spacer domain, a transmembrane region, and a 4-1BB costimulatory signaling domain fused to the cytoplasmic region of the CD3ζ chain. This meso-CAR was successfully expressed on human primary T cells and had antitumor effects *in vitro*. In a subcutaneous injection mouse model, meso-CART cells had a modest inhibitory effect on tumors *in vivo*. More importantly, meso-CART cells completely repressed the development of lung metastases arising from pancreatic cancer, highlighting the powerful antitumor efficacy of meso-CART cells for treating metastatic lesions.

## RESULTS

### Design of meso-CAR gene and expression of meso-CAR in human primary T cells

**Figure 1:**
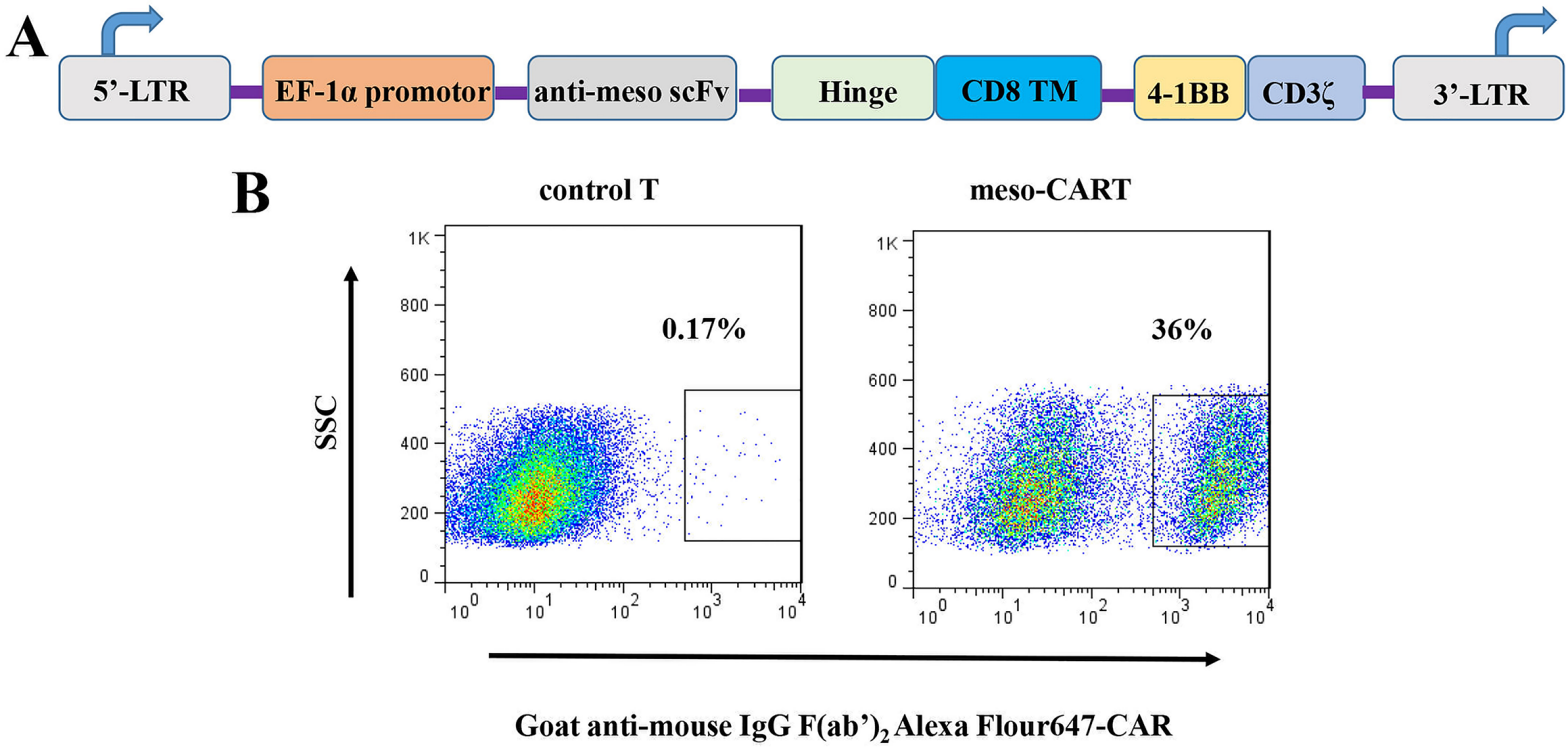
Schematic diagram of meso-CAR gene constructs and meso-CAR expression on T cells after lentivirus transduction (**A**) Diagram of the meso-CAR cassette, which consists of CD8α signal peptide, anti-mesothelin scFv, a spacer domain, a transmembrane region, and a 4-1BB costimulatory signaling domain fused to the cytoplasmic region of the CD3ζ chain. (**B**) Meso-CAR expression was detected with Alexa Fluor 647 goat anti-mouse IgG, F(ab’)_2_ antibody after 7 days transduction. T cells transduced with GFP lentivirus served as controls; meso-CART: T cells transduced with meso-CAR gene.

In this study, we constructed second-generation meso-CART cells containing a 4-1BB co-stimulation domain; its expression was driven by the EF-1α promotor (Figure 1A). Human peripheral blood monocyte cells (PBMCs) from healthy consenting donors were activated and transduced with lentivirus encoding the meso-CAR gene. CAR surface expression in meso-CART cells was examined using flow cytometry with Alexa Fluor 647-conjugated goat anti-mouse IgG, F(ab’)_2_ antibody 7 days after transduction (Figure 1B). Approximately 36% of meso-CART cells were CAR-positive, demonstrating that transduction of lentiviral vectors successfully induced meso-CAR expression in human primary T cells.

### Establishment of mesothelin-expressing pancreatic cancer cells

**Figure 2:**
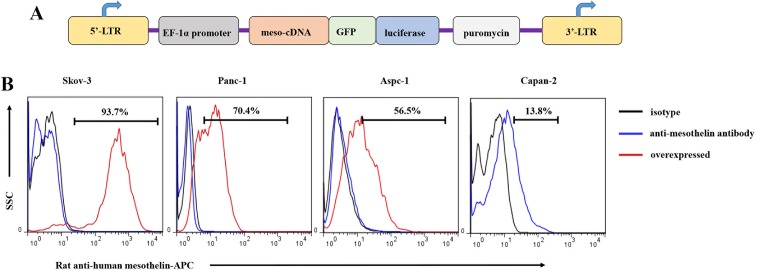
Mesothelin expression in tumor cells and generation of mesothelin+ tumor cell lines (**A**) Diagram of the lentiviral human mesothelin cassette expression vector, which consisted of a full-length human mesothelin antigen, luciferase, and puromycin selection marker. (**B**) Mesothelin expression in various tumor cell lines was measured using rat anti-human mesothelin antibody and flow cytometry. The black bar represents the isotype control, the blue bar represents tumor cell staining with rat anti-human mesothelin antibody, and the red bar represents mesothelin overexpression tumor cells detected with anti-human mesothelin antibody.

To evaluate the ability of meso-CART cells to target tumor cells expressing mesothelin, we constructed a lentiviral vector expressing full-length human mesothelin (Figure 2A) and examined its expression in several tumor cell lines using flow cytometry (Skov-3 human ovarian tumor and Panc-1, Aspc-1, and Capan-2 pancreatic cancer cell lines) (Figure 2B, black line: isotype, blue line: anti-mesothelin antibody). Only 13% of Capan-2 cells naturally expressed mesothelin. We therefore transfected Skov-3, Panc-1, and Aspc-1 cells with the lentiviral mesothelin expression vector (designated Skov3-meso, Panc-1-meso, and Aspc-1-meso, respectively). After lentivirus transduction, transfected cells were selected using 3 μg/mL puromycin over 14 days; mesothelin expression was then examined in selected cells (Figure 2B, red line). MFI values indicated that mesothelin expression was moderate in Panc-1-meso (70%) and Aspc-1-meso (50%) cells, while Skov3-meso cells expressed high levels of mesothelin. These tumor cells were used for subsequent *in vitro* and *in vivo* experiments.

### Characterization of meso-CART cells

**Figure 3:**
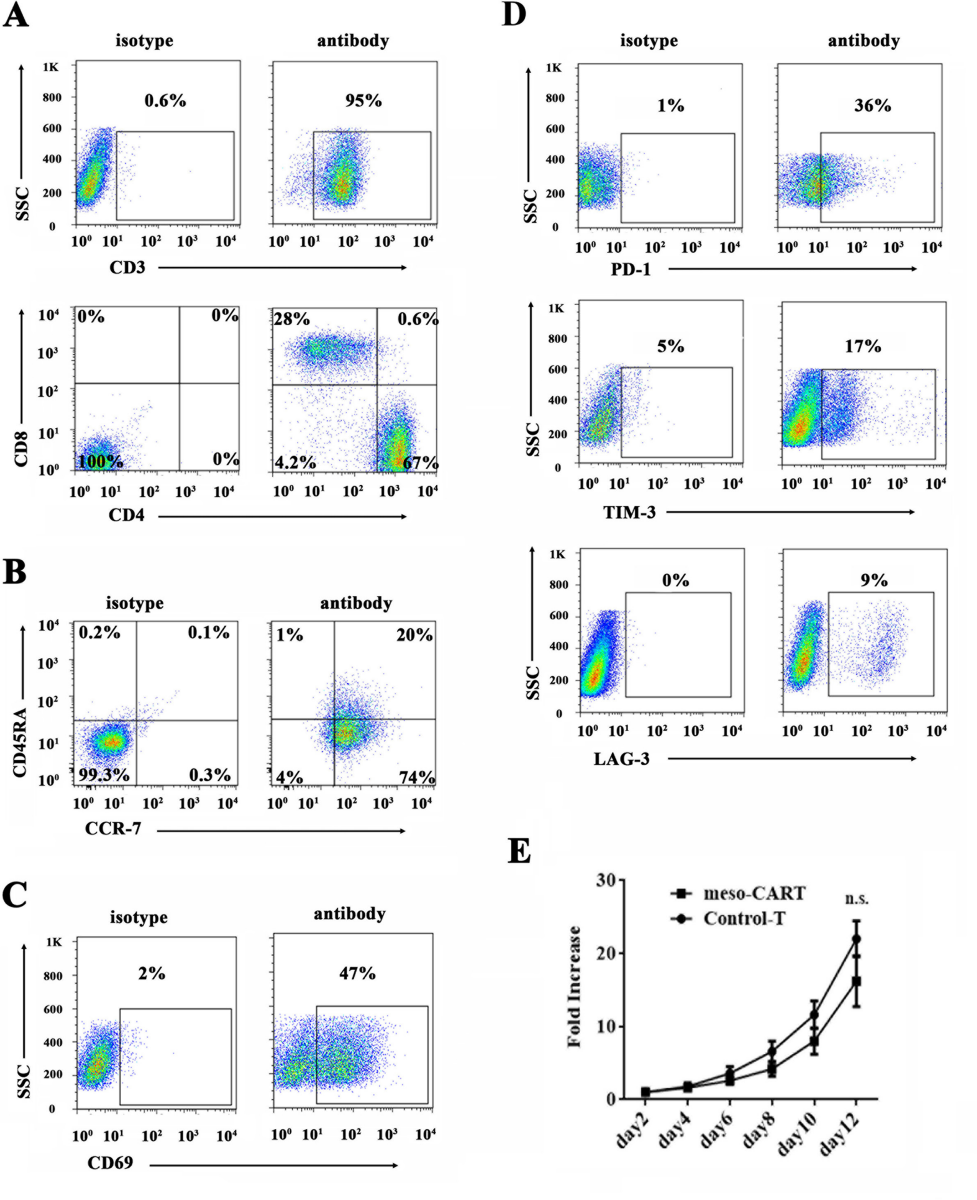
Phenotype and proliferation in T cells transduced with meso-CAR (**A**–**D**) CD3+ cells were the most abundant cell type after 10 days of T cell expansion. On day 10, meso-CART cells were stained with mouse anti-human CD3, CD4, CD8 (A), memory markers CD45RA and CCR-7 (B), activation marker CD69 (C), or exhaustion markers PD-1, LAG-3, and TIM-3 (D) and evaluated using flow cytometry. The flow cytometry data represent all cells in culture. (**E**) Proliferation of meso-CART and GFP-T cells. Data are shown as means ± S.D. n.s.: non-significant difference.

Next, we examined T cell phenotypes 7 days post-transduction (Figure 3A). More than 95% of T cells were CD3^+^, and a majority expressed the CD4^+^ phenotype (67% CD4^+^, and 28% CD8^+^; CD4/CD8 ratio approximately 2:1). Studies indicate that a CD4/CD8 ratio of approximately 1:1 is associated with enhanced treatment efficiency [20]. It was therefore necessary to adjust the CD4^+^:CD8^+^ T cell ratio in this study to increase antitumor efficacy. Meso-CART cells were further analyzed using the differentiation markers CD45RA and CCR-7 (Figure 3B). Most T cells were central memory T (Tcm) cells (CD45RA^+^, CCR-7^-^), while 20% were naive T cells (CD45RA^+^, CCR-7^+^). Next, we detected activation (CD69) and exhaustion (PD-1, LAG-3, TIM-3) markers in the meso-CART cells (Figure 3C and 3D). Approximately 50% of the meso-CART cells were CD69^+^, and expression of all exhaustion markers was lower in meso-CART cells relative to the control cells.

After transduction with the meso-CAR gene, we compared the proliferation characteristics of control T cells and meso-CART cells (Figure 3E). Growth rates were similar in meso-CART and control T cells; after 12 days of culture, the number of non-transduced control T cells increased approximately 22-fold, while meso-CART cell numbers increased approximately 17-fold. These results indicate that transduction of the meso-CAR gene did not impact phenotype or proliferation ability in T cells.

### Meso-CART cells release cytokines and exhibit cytolytic functions when cocultured with mesothelin^+^ tumor cells

**Figure 4:**
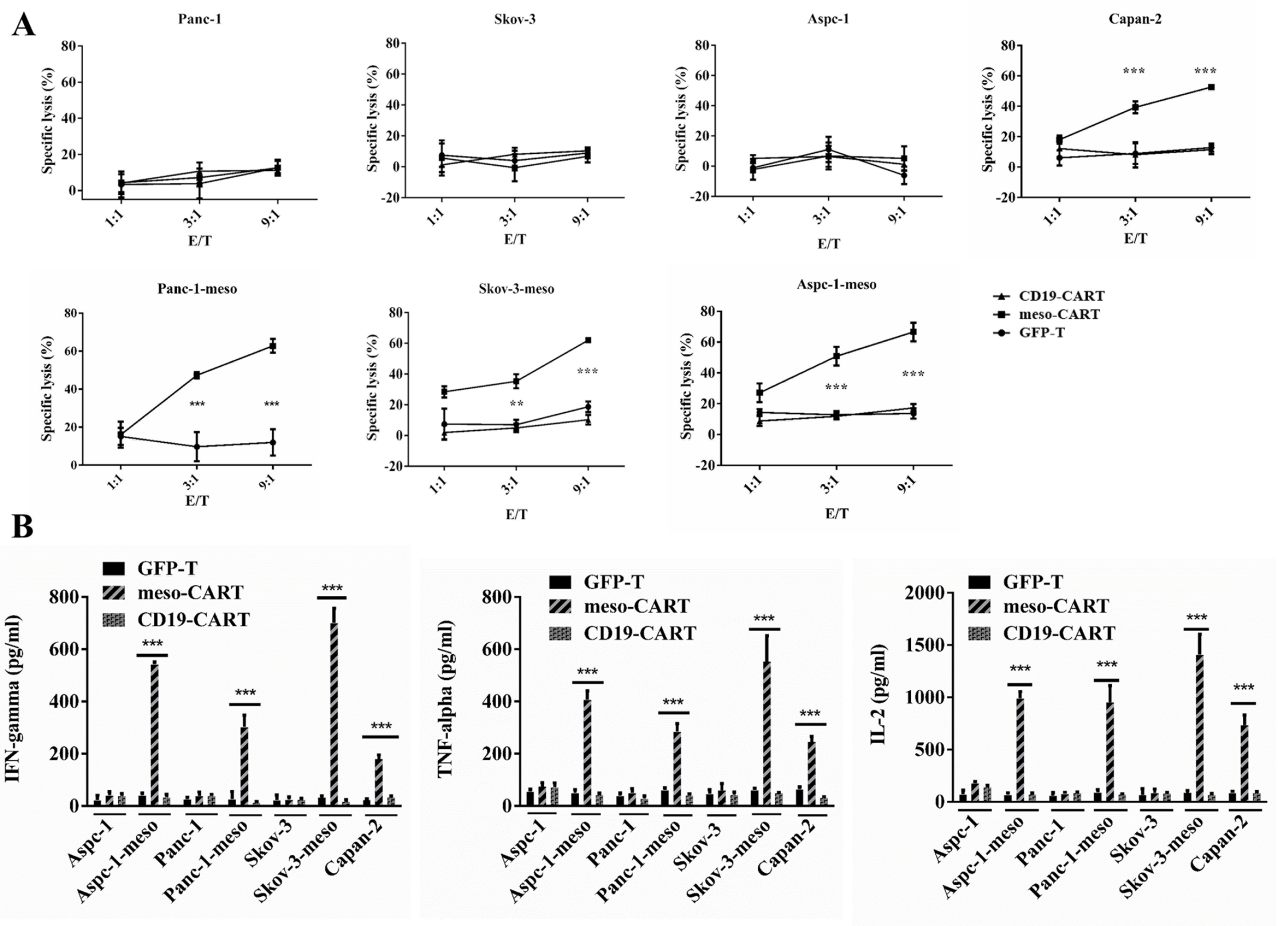
Mesothelin-specific cytotoxicity and cytokine secretion of meso-CART cells (**A**) GFP-T or meso-CART cells were co-cultured with mesothelin^+^ or mesothelin^-^ tumor cells at the indicated effector-to-target (E/T) ratios in a 96-well plate for 16 hours; cytotoxicity was then evaluated using bioluminescence. GFP-T cells incubated with mesothelin^+^ cells and meso-CART cells incubated with mesothelin^-^ tumor cells served as negative controls. (*n* = 3, ^***^p < 0.001). (**B**) Supernatants were collected from the medium after 16 hours of coculture and the ELISA assay was used to determine levels of secreted IL-2, TNF-α, and IFN-γ. Means ± S.D. of triplicate cultures are shown. (*n* = 3, ^***^p < 0.001).

To test whether meso-CART cells were capable of specifically recognizing and causing lysis of mesothelin-expressing tumor cells, we cocultured meso-CART cells, CD19 CART, or GFP-T cells with a panel of tumor cell lines in a 16-hour bioluminescence assay (Figure 4A). Meso-CART cells promoted lysis of mesothelin^+^ Skov3-meso, Panc-1-meso, Aspc-1-meso, and primary Capan-2 cells, but not mesothelin^-^ Aspc-1, Skov-3, or Panc-1 cells. The extent of this lysis was dependent on the effector/target ratio (E/T); as E/T increased, meso-CART cell-induced toxicity in mesothelin^+^ tumor cells increased. The cytotoxicity of meso-CART cells was highest at an E/T of 9:1, at which 70% of all mesothelin^+^ cells were lysed. In contrast, CD19-CART cells and GFP-T cells resulted in very low levels of lysis in both mesothelin^+^ and mesothelin^-^ cell lines.

After tumor cells and meso-CART cells were cultured together for 16 hours, we examined secretion of cytokines associated with the Th1 phenotype (Figure 4B). meso-CART cells secreted high levels of interferon-γ (IFN-γ), interleukin-2 (IL-2), and tumor necrosis factor-α (TNF-α) in response to mesothelin^+^ tumor cells (Aspc-1-meso, Skov3-meso, Panc-1-meso, and Capan-2), but not mesothelin^-^ cells (Aspc-1, Skov-3, and Panc-1). Furthermore, amounts of Th1 cytokines secreted in response to mesothelin^+^ tumor cells were positively correlated with mesothelin expression levels. Control T cells secreted very low levels of these cytokines in response to both tumor cell types. Taken together, these data demonstrate that meso-CART cells recognized and killed mesothelin^+^ cells in a mesothelin-dependent manner and secreted Th1 cytokines in response to mesothelin^+^ tumor cells.

### Antitumor activity of meso-CART cells in subcutaneous pancreatic cancer mouse model

**Figure 5:**
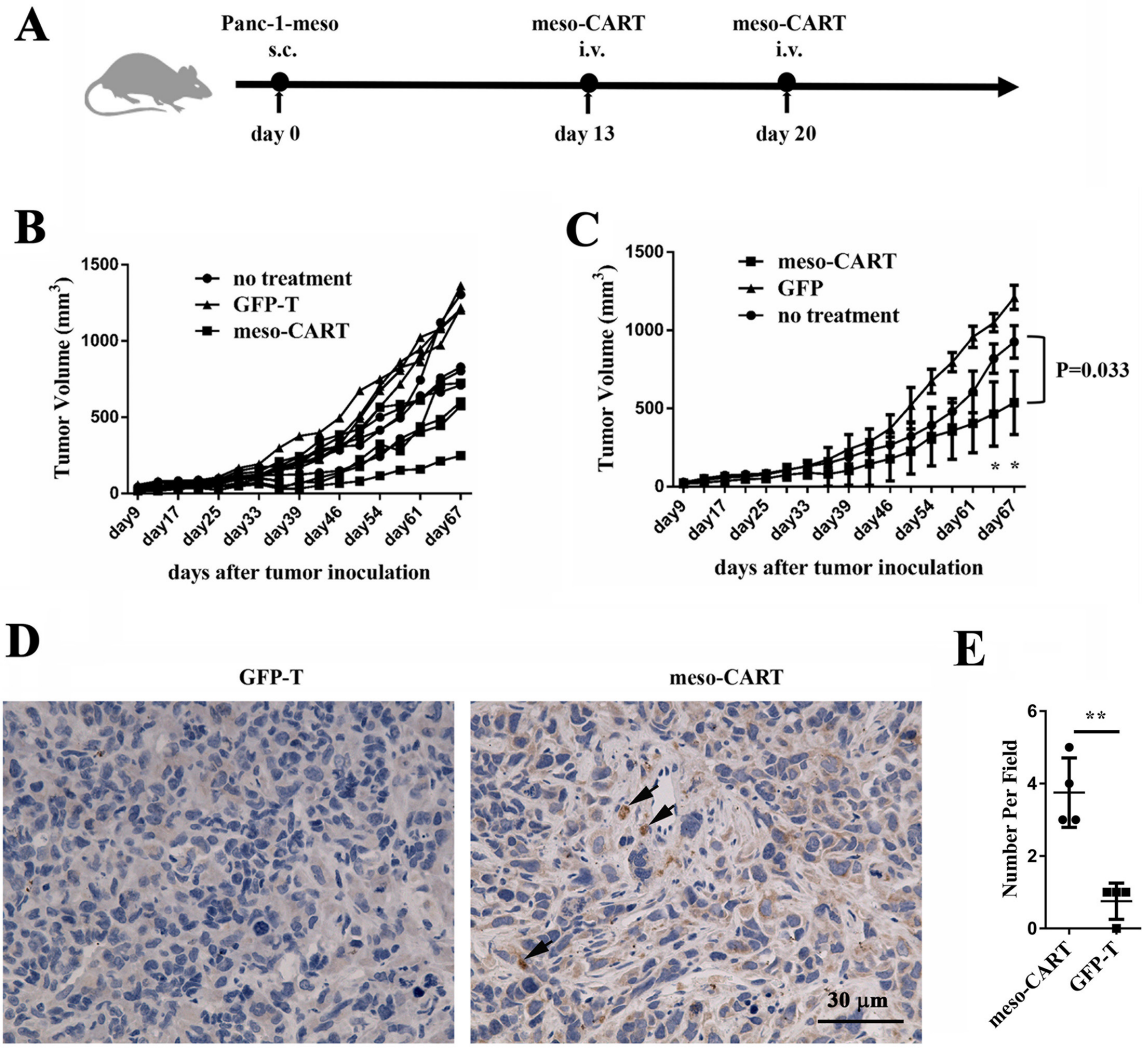
Antitumor efficacy of meso-CART cells in the subcutaneous pancreatic tumor model (**A**) Diagram of the meso-CART cell *in vivo* experiment. (**B**) Tumor growth curves after T or meso-CART cell injection; tumor volumes for all mice were determined twice per week. (**C**) Statistical analysis of tumor volumes after tumor inoculation. All mouse experiments were evaluated using two-way ANOVAs. Error bars represent S.D. (*n* = 6, ^*^p < 0.05). (**D**) At the end of the experiment, tumors were removed and stained with CD3 to examine T cell infiltration. Representative staining from a mouse treated with GFP-T cells and a mouse treated with meso-CART cells is shown. Scale bar, 50 μm. (**E**) Statistical analysis of T cell infiltration in tumor sites. (*n* = 4, ^**^p < 0.01).

To evaluate the antitumor activity of meso-CART cells in pre-established, human-derived tumors *in vivo*, a total 1×10^6^ Panc-1-meso cells were subcutaneously injected into NPG mice. Mice with established subcutaneous Panc-1-meso tumors (approximate volumes 50∼100mm^3^) received meso-CART cell infusions via the tail vein on post-tumor inoculation days 13 and 20 (Figure 5A and 5B). Injections of meso-CART cells moderately decreased tumor volumes compared to those in untreated mice or mice that received GFP-T cells (Figure 5C, *p* = 0.033). At the end of the experiment, the average tumor volume in the group that received meso-CART cells was half of that of the group that received GFP-T cells, indicating that second generation meso-CAR T cells suppress tumor growth *in vivo*. Immunohistochemistry (IHC) analysis of tumors using the T cell marker CD3 was performed to evaluate infiltration of human T cells (Figure 5D and 5E). A modest accumulation of human CD3^+^ T cells (indicated by black arrow) was observed in tumor lesions 9 weeks after intravenous injection of meso-CART cells. However, CD3^+^ T cells were not detected in tumor-bearing mice injected with GFP-T cells. These data indicated that meso-CART cells modestly delayed subcutaneous tumor progression, even though only a small proportion of T cells infiltrated the tumor sites.

### Meso-CART cells suppressed lung metastases in pancreatic cancer

**Figure 6:**
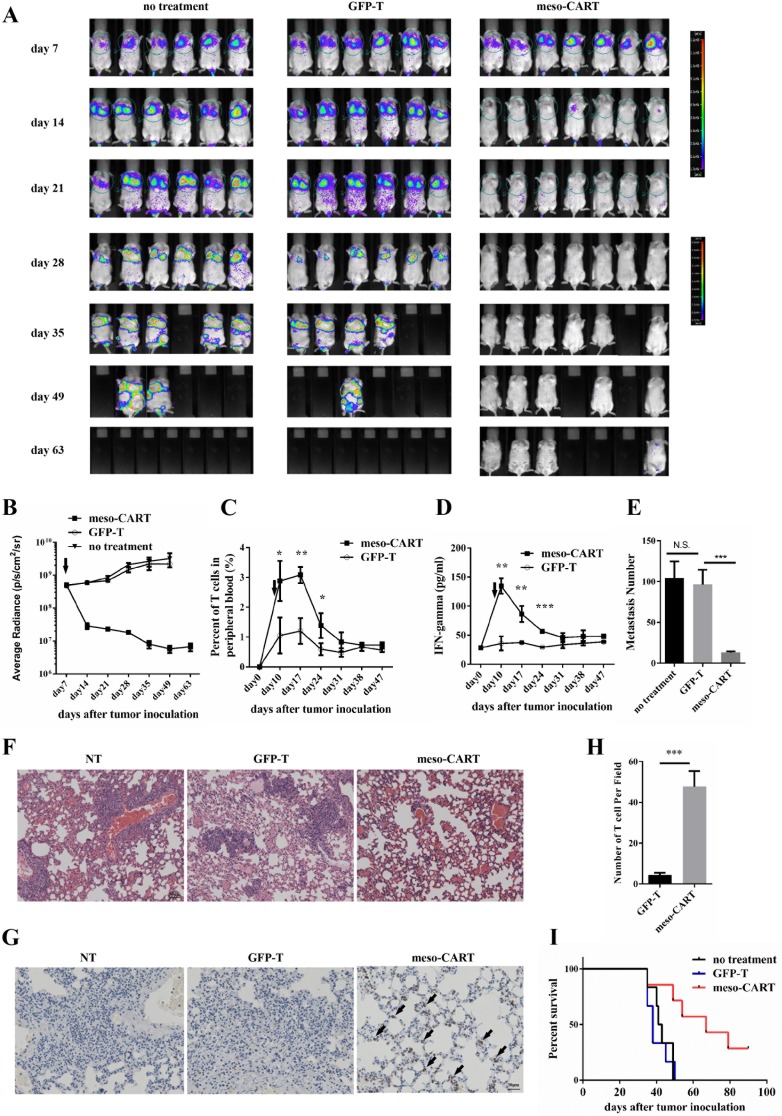
Meso-CART cells inhibit lung metastasis arising from pancreatic cancer 5×10^6^ Panc-1-meso cells were injected via the tail vein in NPG mice on day 0; tumor growth was monitored by BLI once a week. (**A**) Pictures show BLI for NPG mice at different time points after tumor inoculation. (**B**) Graphs of lung tumor growth quantified as photon sec cm sr. Graphs show means ± SD. (^***^p < 0.001). (**C**) T cell percentages in peripheral blood of tumor-bearing mice treated with control T cells or meso-CART cells. (*n* = 3, ^*^p < 0.05, ^**^ *p* < 0.01). (**D**) Secreted IFN-γ levels in peripheral blood after treatment. (*n* = 3, ^**^p < 0.01, ^***^p < 0.001). (**E**) Statistical analysis of metastatic lung nodules in tumor-bearing mice from each group. (**F**) HE staining of lung metastases in the three groups. (**G**) IHC staining of T cells with CD3 antibody in lung metastases from T cell- or meso-CART cell-treated mice. (**H**) Statistical analysis of infiltrated T cells in the lungs. (*n* = 4, ^***^p < 0.001). (**I**) Kaplan-Meier survival analysis of untreated mice and mice treated with T cells or meso-CART cells (*p* < 0.01).

A majority of advanced solid tumors induce metastasis in the lungs, liver, or other organs. We therefore evaluated the efficiency of meso-CART cells in treating lung metastases induced by injection of Panc-1-meso cells via the tail vein in mice. Mice were randomly assigned to groups that received injections of either meso-CART cells or control T cells via the tail vein 7 days after tumor inoculation. Lung metastasis was suppressed 7 days after meso-CART cell injection compared to untreated and control T cell-treated mice (Figure 6A). Meso-CART cell treatment reduced the tumor burden in the lungs compared to the control group (Figure 6B). Mice treated with meso-CART cells remained tumor-free without a detectable increase in bioluminescence. However, 49 days later, some meso-CART cell-treated mice had died even though tumor burdens had not increased (Figure 6A). Additional studies are therefore needed to determine whether the mice died due to side effects of CART cell implantation or due to the migration and proliferation of mesothelin^-^ tumor cells. CD3^+^ T cells were detected in the peripheral blood of mice treated with meso-CART cells and persisted for approximately 20 days *in vivo*. In this group, T cell levels slowly decreased during regression of metastatic lung tumors. In contrast, CD3^+^ T cells were not detected in the peripheral blood of control group mice (Figure 6C). In addition, IFN-γ secretion in the peripheral blood was increased 3 days after meso-CART cell injection compared to the control groups (Figure 6D); IFN-γ levels decreased as clearance of the tumor progressed. At the end of the experiment, which occurred when mice became distressed and moribund, the mice were sacrificed and numbers of metastatic lung nodules were counted. On average, nearly 100 metastatic lung nodules were observed in untreated and GFP-T cell-treated mice; meso-CART cell implantation dramatically suppressed the lung metastasis to an average of approximately 10 nodules (Figure 6E). Accordingly, more metastasis sites were detected by HE staining in the control group mice (Figure 6F). To determine why CART cell therapy was more effective against lung metastasis than against s.c. primary tumors, infiltration of T cells into the lungs was assessed using IHC with a CD3 antibody. As shown in Figure 6G and Figure 6H, more CD3+ T cells infiltrated the lungs of mice treated with meso-CART cells, while CD3+ T cells were nearly absent in the lungs of control group mice. In addition, meso-CART cell treatment prolonged survival rates compared to control group mice (Figure 6I). Together, these preclinical data indicate that systemically administered meso-CART cells suppress lung metastases arising from advanced pancreatic tumors.

## DISCUSSION

In this study, we found that meso-CART cells killed pancreatic cancer cells *in vitro* and repressed subcutaneous tumor growth *in vivo*. In addition, meso-CART cells rapidly and eliminated lung metastases in all treated animals. Previous studies focusing mainly on the use of meso-CART cells to treat subcutaneous or intraperitoneal (i.p.) human-derived tumors have found that these cells have relatively limited anti-tumor effects. Here, we demonstrate that meso-CART cells successfully inhibited lung metastases originating from pancreatic cancer; these cells might therefore be an effective clinical treatment for patients with lung metastases.

CAR can be designed to incorporate different costimulatory molecules, such as 4-1BB, CD27, CD28, and OX-40 [21–23]. These costimulatory molecules have different functions and can influence activation threshold, response type, survival time, and phenotype in CART cells [24]. The expression of exhaustion markers (PD-1, LAG-3, TIM-3) is reduced in second-generation CART cells containing a 4-1BB domain in the presence of tumor antigen. In addition, these CART cells persisted longer *in vivo* than CART cells containing a CD28 domain [25]. In this study, we constructed second-generation CART cells with a 4-1BB costimulatory domain that targets mesothelin. Expression of exhaustion markers was also reduced in these meso-CART cells. Long-term persistence of CART cells *in vivo* is crucial to their antitumor efficacy [16]. Here, the meso-CART cells proliferated and persisted in peripheral blood for about 3 weeks in a systemic tumor model, which was long enough to eliminate the tumor. The persistence of these meso-CART cells was also associated with prolonged IFN-γ secretion, which has antitumor effects.

The antitumor efficacy of CART cells may differ depending on T cell type, and CART cells with the Tcm phenotype might be most effective for adoptive cell therapy [26]. In addition, co-culture with IL-7 and IL-15 might produce a higher proportion of CART cells with the Tcm phenotype [27]. In this present study, we found that a majority of meso-CART cells had the Tcm phenotype, even though they were cultured with IL-2. The antitumor efficacy and long-term persistence of these meso-CART cells might be a result of this Tcm phenotype. In addition, CD4 and CD8 expression status in T cells may also affect the antitumor activity, proliferation, and persistence of CART cells *in vivo* [20]. A combination of CD4^+^ and CD8^+^ T cells enhanced antitumor activity compared to the use of cytotoxic CD8+ T cells alone [28]. Moreover, naive CD4+ T cells and Tcm CD8^+^ T cell subsets were the most potent *in vivo*. The CD4/CD8 ratio for the meso-CART cells generated here was 2:1; this may be the ideal ratio and likely contributed to the antitumor effects of these meso-CART cells.

Interestingly, in this study, a single lower-dose injection of meso-CART cells (E/T of 1:1) largely eradicated metastatic lung tumors in a systemic pancreatic cancer tumor model. In most relevant clinical trials, CART cells were injected in a dose-acceleration manner due to safety concerns [29, 30]. Our results suggest that a single, low E/T ratio dose of CART cells might not only be ideal for pancreatic cancer patients with lung metastasis, but might also reduce potential side effects. In addition, the systemic tumor model developed here might be useful for further evaluating the function of CART cells *in vivo*, especially for solid tumors with distant metastases in the lungs.

One important limitation of the application of CART cells in clinical trials is the lack of appropriate tumor-specific antigen targets [31]. Mesothelin is a tumor-associated antigen that is highly expressed in many tumors, including ovarian cancer, lung adenocarcinoma, and pancreatic adenocarcinoma. It plays pivotal roles in tumor cell proliferation, angiogenesis, metastasis, and tumor occurrence. In this study, T cells in which meso-CAR expression was induced were able to eliminate metastases arising from pancreatic cancer, indicating that mesothelin is an effective target for the treatment of late-stage, malignant metastatic tumors. However, mesothelin is also expressed in normal tissue [32]. Meso-CART cells may therefore bind to normal tissue, potentially causing toxicity. The expression of suicide genes, such as inducible Caspase9, together with the dimerizing drug AP1903 could be used to destroy the CART cells and thus reduce any side effects.

Thus far, clinical response rates for the treatment of solid tumors with CART cells have been lower than rates observed in melanoma and hematologic malignancies, and many patients with solid tumors fail to respond to CART cell therapy. In this study, the subcutaneous model was established using only tumor cells without any stromal cells; the structure of resulting tumors was so compact that meso-CART cells might have been unable to infiltrate the tumors, bind to the mesothelin antigen, proliferate, and kill the cancer cells at an early stage. In later stages, specifically during the last two days of monitoring, CART cells did inhibit tumor growth. Despite the small initial volume of the subcutaneous tumors (approximately 50mm^3^) at the time of meso-CART cell treatment, meso-CART cells merely delayed tumor progression rather than eliminating the primary tumor. Differences in the efficacy of meso-CART cell treatments in lung metastasis and subcutaneous tumors might be a result of the differing CART cell infiltration frequencies, as characterized by CD3^+^ T cell staining in tumor lesions; more CD3^+^ T cells infiltrated and accumulated in lung metastases compared to subcutaneous tumors (Figure 5D and Figure 6G). This observation suggests that it is difficult for meso-CART cells to migrate to and infiltrate solid tumors. A combination of obstacles, including tumor heterogeneity, immunosuppressive tumor microenvironment, and limited infiltration makes it difficult for CART cells to act on solid tumors. Our results indicate that meso-CART cells were more effective at migrating to and invading distant metastases. Further manipulations, perhaps including co-expression of the chemokine receptor CCR2b (ligand for CCL2) to enhance the infiltration of CART cells [33, 34] or co-expression of the enzyme heparanase (HPSE) [35] to degrade components of the extracellular matrix, may be necessary to effectively treat compact primary tumors with CART cells.

In summary, we have constructed meso-CART cells that secrete cytokines and exert cytotoxic effects in response to mesothelin-positive tumor cells both *in vitro* and *in vivo*. These meso-CART cells eliminated metastatic lung nodules originating from pancreatic cancer and delayed subcutaneous pancreatic tumor growth, indicating that meso-CART cells may be an effective treatment for patients with distant lung metastasis.

## MATERIALS AND METHODS

### Generation of lentivirus vector and lentiviral particle

The mesothelin-specific single-chain fragment variable (scFv, clone number SS1) was synthesized by BGI Genomics (Beijing, China). The meso-CAR included human CD8α signal peptide, the scFv, the spacer domain (Hinge) and transmembrane domain of CD8a, a 4-1BB co-stimulation molecule, and a CD3ζ domain. All components were inserted into a pRRL lentivirus vector. Full-length human mesothelin cDNA was also cloned into a pRRL lentivirus vector to generate Skov-3 and Panc-1 cells that overexpressed mesothelin. Replication-defective lentivirus supernatant was produced as follows: 8×10^6^ HEK-293T cells in a 10-cm dish were co-transfected with 16 μg of lentivirus vector DNA, 12 μg of packaging vector DNA (pSPAX_2_), and 4 μg of envelope DNA (pMD2.G). The medium was changed to fresh high glucose DMEM medium (Gibco, USA) with 10% fetal bovine serum (FBS) (HyClone, Logan, UT) after 12 hours. The lentivirus particle supernatant was collected 48 and 72 hours post-transduction. Cell debris was removed by centrifugation at 3000 rpm for 10 minutes then filtered through 0.22 μm polyvinylidene fluoride filters (Millipore, USA). The lentiviral supernatant was stored at –80°C.

### Cell lines

The Panc-1, Aspc-1, and Capan-2 pancreatic cancer cell lines, the Skov-3 ovarian adenocarcinoma cell line, and human HEK-293T cells were obtained from the National Infrastructure of Cell Line Resource (Beijing, China) and cultivated in high glucose DMEM medium (Gibco, USA) plus 10% FBS supplemented with 2mM GlutaMAX-I (Gibco, USA) and penicillin/streptomycin (Gibco, USA).

### Isolation, activation, and lentiviral infection of human T cells

All Peripheral Blood Mononuclear Cells (PBMCs) used in our study were obtained from healthy donors who provided informed consent (Blood Center of Beijing Red Cross Society). Human T lymphocytes were cultured in RMPI-1640 medium (Gibco, USA) supplemented with 10% FBS and 300 U/mL interleukin-2 (Peprotech, USA). After 48 hours of activation with anti-human CD3/CD28 Dynabeads (Gibco, USA), 8 μg/mL polybrene (Millipore, USA) and 300 U/mL IL-2 were added to each well and T cells were transduced twice over the next 48 hours with meso-CAR lentivirus by spinoculation for 1 hour. Transduction efficiency was determined by flow cytometry 7 days later.

### Lentiviral transduction of Skov-3, Aspc-1, and Panc-1 cells with mesothelin-GFP-luciferase gene

Skov-3 human ovarian cancer and Aspc-1 and Panc-1 pancreatic cancer cells were transduced with a lentiviral vector containing full-length human mesothelin, luciferase, and a GFP cassette driven by an EF-1α promoter. Skov-3, Aspc-1, and Panc-1 cells were transduced two more times with the lentivirus over the next 48 hours; these cells are referred to as Skov-3-meso, Aspc-1-meso, and Panc-1-meso cells. After 2 days of transduction, Skov-3-meso, Aspc-1-meso, and Panc-1-meso tumor cells were sorted with FACSAria based on GFP expression and were then screened by two weeks of treatment with 3 μg/mL puromycin (InvivoGen, USA). Human mesothelin expression in the tumor cells was evaluated by flow cytometry using rat anti-human mesothelin-APC antibody (RD, USA).

### Flow cytometry

We used a FACSCalibur instrument and Cellquest software for all flow cytometry analysis. All cells in culture were washed with PBS before staining with antibodies. After 30 to 45 min of incubation at 4°C in the dark, cells were washed twice with PBS (Gibco, USA) and analyzed on the FACSCalibur instrument. Meso-CAR expression was monitored by flow cytometry using the Alexa Fluor 647-conjugated F(ab’)_2_ antibody (Jackson Immunology, USA) directed against the scFv. Stained, un-transduced T cells or isotype antibodies were used as controls. Anti-human CD3, CD4, CD8, CD69, LAG-3, TIM-3, and PD-1 (CD279) antibodies were purchased from BD.

To measure T cells in mouse blood, 100 μL of venous blood was obtained weekly from the orbital venous plexus and stained with anti-human CD3 antibody. Flow cytometry data were analyzed using FlowJo software.

### Cytotoxicity assay

The ability of meso-CART cells to lyse target tumor cells was measured using a bioluminescence assay as previously described [36]. In these assays, meso-CART or GFP-T cells were co-incubated with luciferase-expressing target cells at various effector-to-target ratios (E:T) in RMPI-1640 medium in 96-well U-bottom plates for 16 hours. Lysis percentage was determined using the following equation: % specific lysis = 100 × (spontaneous death RLU – test RLU) / (spontaneous death RLU). RLU: relative light units.

### Cytokine release assay

Secretion of cytokines by meso-CART cells was examined in Skov-3-meso and Panc-1-meso tumor cells. Briefly, effector cells (1 × 10^5^) were co-cultured with tumor cells in RMPI-1640 medium at a final volume of 200 μL in 96-well plates. The supernatants were harvested 16 hours after co-culture and IFN-γ, IL-2, and TNF-α levels were measured by ELISA (DAKAWE).

### Animals

Severe combined immune-deficient NPG mice (members of the NOD-Prkdc^scid^ IL-2rg^null^ family) were obtained from Vitalstar Biotechnology Co., Ltd. (Beijing, China) and bred under specific pathogen-free conditions at Peking University Health Science Center. Institutional Animal Care and Use Committee approval was obtained from the Peking University Health Science Center. Healthy female mice between 6–8 weeks of age were randomly assigned to the experimental or control groups. For the subcutaneous tumor mouse model, tumor volumes were determined twice a week using the following formula: V = 1/2 × (length × width × width). Mice that developed hind-limb paralysis, which indicated tumor progression, were sacrificed.

### *In vivo* testing of antitumor activity of meso-CART cells

In the subcutaneous tumor mouse model, 1×10^6^ Panc-1-meso cells suspended in 200 μL PBS were subcutaneously injected into the flanks of NPG mice. After tumors (approximately 50–100mm^3^ in size) were established (usually after 2 weeks), the mice were randomly assigned to experimental groups and received either no treatment or ten million GFP-T or meso-CART cells via the tail vein; the same treatment was repeated 7 days later. Treatment efficacy was evaluated by examining infiltration of CD3^+^ T cells. For the intravenously injected tumor mouse model, 5 × 10^6^ Panc-1-meso cells were infused via the tail vein; 1 week later, mice were injected with either 5 × 10^6^ meso-CART cells or control T cells via the tail vein. Tumor burden was monitored weekly using the Xenogen IVIS (Caliper Life Sciences). Mice were injected intraperitoneally with 150 mg/kg D-luciferin (GoldBio, USA) and imaged 10 minutes later. The therapeutic effects of meso-CART cell treatment were evaluated based on bioluminescence signals.

### Statistical methods

All data are shown as means ± S.D. Data were analyzed using GraphPad Prism. Student’s *t*-test was used to evaluate differences in cytokine secretion and specific cytolysis. *P*-values < 0.05 were considered significant.
